# Fluvastatin Upregulates the *α*_1C_ Subunit of CaV1.2 Channel Expression in Vascular Smooth Muscle Cells via RhoA and ERK/p38 MAPK Pathways

**DOI:** 10.1155/2014/237067

**Published:** 2014-12-30

**Authors:** Qiu-Fang Ouyang, Ying Han, Zhi-Hong Lin, Hong Xie, Chang-Sheng Xu, Liang-Di Xie

**Affiliations:** ^1^Fujian Hypertension Research Institute, The First Affiliated Hospital of Fujian Medical University, Fujian 350005, China; ^2^Ultrasound Department, The Second Affiliated People's Hospital, Fujian University of Traditional Chinese Medicine, Fuzhou, Fujian 350003, China

## Abstract

Abnormal phenotypic switch of vascular smooth muscle cell (VSMC) is a hallmark of vascular disorders such as atherosclerosis and restenosis. And this process has been related to remodeling of L-type calcium channel (LTCC). We attempted to investigate whether fluvastatin has any effect on VSMC proliferation and LTCC*α*_1C_ subunit (LTCC*α*_1C_) expression as well as the potential mechanisms involved. The VSMCs proliferation was assayed by osteopontin immunofluorescent staining and [^3^H]-thymidine incorporation. The cell cycle was detected by flow cytometric analysis. The activity of RhoA was determined with pull-down assay. MAPK activity and LTCC*α*_1C_ expression were assessed by western blotting. We demonstrated fluvastatin prevented the VSMCs dedifferentiating into a proliferative phenotype and induced cell cycle arrest in the G0/G1 phase in response to PDGF-BB stimulation. Fluvastatin dose-dependently reversed the downregulation of LTCC*α*_1C_ expression induced by PDGF-BB. Inhibition of ROCK, ERK, or p38 MAPK activation largely enhanced the upregulation effect of fluvastatin (*P* < 0.01). However, blockade of JNK pathway had no effect on LTCC*α*_1C_ expression. We concluded LTCC*α*_1C_ was a VSMC contractile phenotype marker gene. Fluvastatin upregulated LTCC*α*_1C_ expression, at least in part, by inhibiting ROCK, ERK1/2, and p38 MAPK activation. Fluvastatin may be a potential candidate for preventing or treating vascular diseases.

## 1. Introduction

Vascular smooth muscle cells (VSMCs) show the unique ability of undergoing a well-known phenotypic switch from differentiated, contractile cells to a proliferating phenotype, a process essential for restenosis, atherosclerosis, and hypertension. VSMCs normally exist in a quiescent, differentiated state in the blood vessel wall expressing a unique repertoire of contractile proteins, calcium ion channels, and signaling molecules that are necessary for their contractile properties [1]. Under the pathological conditions, VSMCs lose the contractile phenotype, which is associated with the silencing of contractile marker gene expression and the upregulation of genes that facilitates other cellular functions, such as proliferation and migration [2].

L-type calcium channel (LTCC) serves as a critical pathway gating the influx of Ca^2+^ into the cytoplasm from the extracellular space. LTCC are heteroligomeric complexes consisting of 5 subunits (*α*_1_, *α*_2_, *β*, *γ*, and *δ*). The LTCC *α*_1C_ (LTCC*α*_1C_) subunit functions as a voltage sensor, drug receptor, and Ca^2+^-selective pore. An increase in [Ca^2+^]_i_ initiates VSMCs contraction, which is a key process of excitation-contraction coupling [3]. Convincing data suggests that cell proliferation abolishes the expression of LTCC and reexpression of LTCC*α*_1C_ parallels the reappearance of contractile phenotype marker [4–6]. Substantial evidence shows that platelet derived growth factor (PDGF) induces VSMCs proliferation through the mitogen-activated protein kinase (MAPK) and the Rho associated protein kinase (ROCK) pathways [7]. However, there is a paucity of information available on the specific signal pathway involved in the PDGF-mediated regulation of LTCC*α*_1C_ expression.

It is well known that statins, HMG-CoA (3-hydroxy-3-methyl-glutaryl coenzyme A) reductase inhibitors, exert pleiotropic properties. And they can inhibit VSMCs proliferation [8]. Wagner et al. reported lovastatin could induce VSMC differentiation and prevented the downregulation of contractile protein expression [9]. And our previous studies indicated fluvastatin regressed resistant vessel remodeling, ameliorated vasodilatation function in rats [10], and inhibited VSMCs migration [11]. However, the direct evidence regarding the effects of fluvastatin on the expression of LTCC*α*_1C_ in VSMCs and the exact mechanism involved is still unavailable. Therefore, we investigate the effect of fluvastatin on LTCC*α*_1C_ expression in response to PDGF and further explore the potential underlying mechanisms.

## 2. Materials and Methods

### 2.1. Cell Culture and Treatment

VSMCs were isolated from the thoracic aortas of spontaneously hypertensive rats by the modified explant technique of Champbell in our laboratory as described previously [12–14]. And the experimental procedures were approved by the Animal Care and Use Committee of Fujian Medical University. Briefly, cells were grown in DMEM (Dulbecco's modified Eagle medium). VSMCs were identified by immunostaining procedures using anti-smooth muscle *α*-actin antibody. And the third passage cells were used for experiments. When cells reached 80%–90% confluence, they were put to serum-free starvation for 24 h to synchronize the cell cycle. To study the time-response of Flu on proliferation and LTCC*α*_1C_ expression, quiescent VSMCs were incubated with 10 *μ*g/L PDGF-BB, in the absence or in the presence of 10^−5^ M fluvastatin. Then VSMCs were collected every 4 h till 24 h. To investigate the dose response of Flu on cell proliferation and LTCC*α*_1C_ expression, VSMCs were preincubated with Flu (gift from Novartis Pharma AG, Switzerland) at the graded concentrations (10^−4^ M~10^−8^ M) for 0.5 h before the addition of PDGF and incubated for 24 h. To determine the role of MAPK pathways in PDGF-mediated effects on LTCC*α*_1C_ expression, confluent quiescent VSMCs were pretreated with the specific ERK1/2 inhibitor (PD98059, 20 *μ*mol/L), p38 MAPK inhibitor (SB203580, 10 *μ*mol/L), JNK inhibitor (SP600125, 50 *μ*mol/L), or ROCK-I/II inhibitor (Y27632, 10 *μ*mol/L) for 1 h, and/or Flu (10^−5^ M) for 0.5 h before the addition of PDGF and incubated for 24 h. To test whether Flu affected the MAPK activity in response to PDGF, VSMCs were cultured with or without fluvastatin (10^−5^ M) for 24 h and then treated with PDGF (10 *μ*g/L) for 5, 15, 30, and 60 min.

### 2.2. Immunofluorescent Staining for Osteopontin and RhoA Expression as well as [^3^H]-thymidine(TdR) Incorporation Analysis for Proliferation in VSMCs

Considering osteopontin was a biomarker of proliferation, osteopontin in VSMCs (1 : 200, Abcam) was detected with immunofluorescent staining. Meanwhile, for localization of RhoA (ras homolog family member A) expression, immunofluorescence for the anti-RhoA (1 : 100; Abcam) was performed in VSMCs. Additionally, cell proliferation was evaluated by [^3^H]-thymidine incorporation to determine DNA synthesis. 1 *μ*Ci/ml [^3^H]-thymidine (Amersham) was added to each well. Incorporated radioactivity was measured using a Betaplate scintillation counter.

### 2.3. Flow Cytometry Analysis of Cell Cycle

Cells density was adjusted to 1.0 × 10^6^ cells/cm^3^ and preincubated with fluvastatin (10^−5^ M) for 0.5 h before the addition of PDGF-BB (10 *µ*g/L) and incubated for 24 h. Cells incubated with PDGF-BB alone served as control. Then the cells were harvested and fixed with 70% ethanol and incubated with RNase A (20 mg/L) and propidium iodide (50 mg/L) for 1 h in the dark. The stained cells were determined using a flow cytometer in combination with Flow Jo software.

### 2.4. Western Blotting Analysis for the Activity of MAPK and LTCC*α*_1C_ Protein Expression

VSMCs were lysed in RIPA buffer (50 mM Tris-Cl, pH 8.0, 150 mM NaCl, 1% Nonidet P-40, 0.5% sodium deoxycholate, 0.1% SDS, 100 *µ*g/mL phenylmethyl-sulfonyl fluoride, and 2 *µ*g/mL aprotinin). The suspension was incubated on ice and then centrifuged (14 000 g, 10 minutes, 4°C). Protein concentrations were measured by Bradford assay with bovine serum albumin as a standard. 60 *µ*g protein supernatants were used for electrophoresis. Primary antibodies were used at the indicated dilutions as follows: LTCC*α*_1C_, 1 : 200 (Alomone); *β*-actin, 1 : 1000; anti-p-p38 MAPK, 1 : 1000; anti-total-p38 MAPK, 1 : 1000; anti-p-JNK, 1 : 1000; anti-total-JNK 1 : 1000; anti-p-ERK1/2 (1 : 1000); and anti-total-ERK1/2 (1 : 1000); all were from Cell Signalling Technology (Danvers, MA). The intensity of the bands was quantified by densitometry. Blots were representative of at least three experiments.

### 2.5. RhoA Pull-Down Assay

Active RhoA was isolated using the Rho activity binding domain of rhotekin as described by Ren and Schwartz [15]. Briefly, cells were preincubated with fluvastatin (10^−5^ M) for 0.5 h before the addition of PDGF-BB (10 *µ*g/L) and treated for 24 h. Then those cells were lysed with buffers and incubated with 40 *µ*g of GST-RBD (Cytoskeleton, Acoma Street, Denver, USA) for 1 hour. After binding, the samples were washed with lysis buffer three times. Pulled-down proteins that are activated Rho (GTP-bound Rho) were fractionated on 12% SDS-PAGE and immunoblotted with polyclonal Ab against RhoA (Santa Cruz Biotechnology, CA, USA). The total cell lysates were also blotted with Ab for RhoA as a loading control. The level of activated RhoA was determined after normalization with the total RhoA present in the same cell lysates.

### 2.6. Statistical Analysis

All data was expressed as the mean ± SEM unless otherwise indicated. Group comparisons were performed with Student's *t*-test (2-sample test) or one-way analysis of variance using SPSS 13.0. All reported probability values were 2-tailed, and a *P* value of 0.05 was considered as statistical significance.

## 3. Results

### 3.1. Fluvastatin Inhibited the Phenotype Switching and Proliferation of VSMC Elicited by PDGF

**Figure 1:**
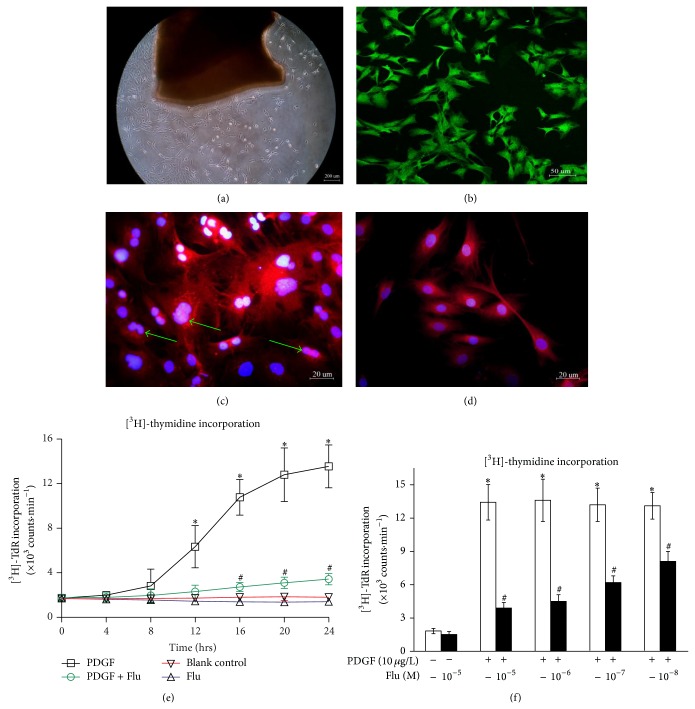
Effects of Flu on VSMCs proliferation analyzed by immunofluorescent staining and [^3^H]-thymidine uptake. Primary cell culture (a); expression of α-smooth muscle-actin in VSMCs as depicted by immunofluorescent staining (b). Enhanced osteopontin expression, nuclear division (arrow), and increased cell density were noted in VSMCs treated with PDGF (c). Reduced osteopontin fluorescence, spindle-shape appearance was observed in VSMCs by coincubation of fluvastatin and PDGF (d). Flu inhibited [^3^H]-thymidine incorporation in a time- and concentration-dependent manner (e, f). Results represented as mean ± SEM of 3 independent experiments in triplicate. ^*^ *P* < 0.05 versus blank control and ^#^ *P* < 0.05 versus cells incubation with PDGF. Flu: fluvastatin; PDGF: platelet derived growth factor; and M: mol/L.

VSMCs were adherently cultured using Petri dishes (Figure 1(a)). As depicted by immunofluorescent staining, *α*-smooth muscle-actin was positive, suggesting those cells were smooth muscle cells (Figure 1(b)). Alternatively, the fluorescence intensity of osteopontin (proliferate phenotype marker) was markedly enhanced in VSMCs treated with PDGF for 24 hs (Figure 1(c)), while it was attenuated by being cocultured with PDGF and 10^−5^ M Flu (Figure 1(d)). Strikingly, VSMCs exposed to PDGF exhibited mostly flattened, fibroblastic appearance, nuclear division, and increased cell density. However, VSMCs cotreated with fluvastatin and PDGF were characterized by spindle-shape morphology and by reduced cell numbers.

Cell proliferation was further confirmed by [^3^H]-thymidine DNA incorporation. PDGF caused time-dependently a substantial increase in [^3^H]-thymidine incorporation in VSMCs, which reached the maximum at the time point of 24 h. It was increased by 6.8-fold compared to blank controls (*P* < 0.05, Figure 1(e)). And this effect was prevented by coadministration of 10^−5^ M Flu and PDGF. Conversely, [^3^H]-thymidine uptake changed insignificantly by fluvastatin treatment, as compared to blank controls.

Additionally, in the presence of PDGF, Flu resulted in a dose-dependent inhibition of VSMC proliferation at the graded concentrations from 10^−5^ M to 10^−8^ M (Figure 1(f)). The lowest [^3^H]-thymidine incorporation was (3.9 ± 0.5) × 10^3^ counts·min^−1^ when Flu concentration was 10^−5^ M. And in the absence of PDGF, there were no significant changes in [^3^H]-thymidine incorporation even when Flu was administrated at the concentration of 10^−5^ M. VSMCs apoptosis was observed when the concentration of Flu ranged from 10^−4^ M to 10^−2^ M (data not shown).

### 3.2. Fluvastatin Induced Cell Cycle Arrest in VSMCs Stimulated by PDGF

**Figure 2:**
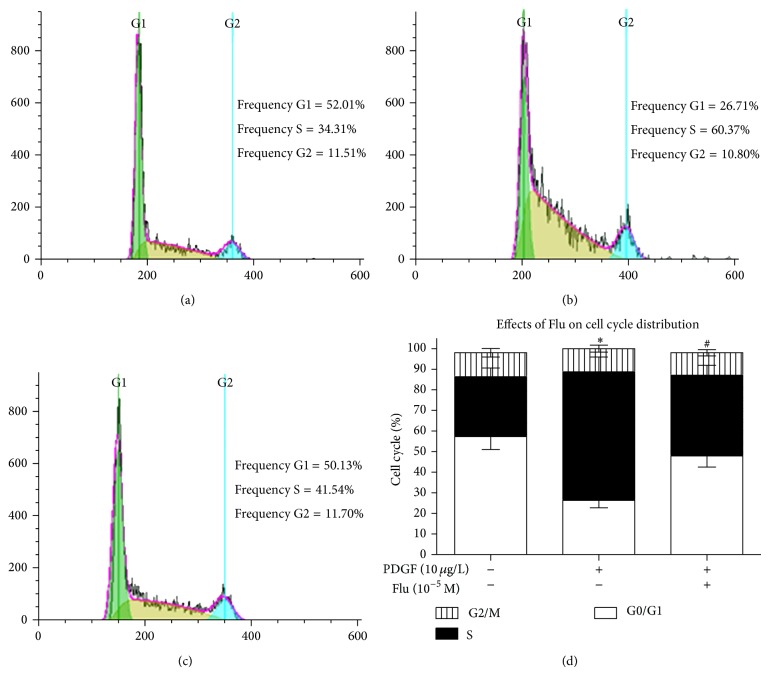
Fluvastatin inhibits cell cycle progression as detected by flow cytometry. VSMCs were incubated for 24 hrs with vehicle (a), PDGF (b), or Flu and PDGF (c). Bar graph illustrating the percentage of cells in G0/G1, S, and G2/M phase by the indicated medication (d). Results represented as mean ± SEM of 3 independent experiments in triplicate. ^*^ *P* < 0.05 versus blank control. ^#^ *P* < 0.05 versus PDGF stimulated cells. Flu: fluvastatin; PDGF: platelet derived growth factor.

The proportion of VSMCs in the G0/G1 phase was decreased when incubated with 10 *µ*g/L PDGF for 24 h (33.8% ± 3.2% versus 66.7% ± 3.8% in vehicle VSMCs). Meanwhile, the percentage of cells in the S phase was increased in the PDGF incubation cells (49.2% ± 2.1% versus 29.6% ± 1.0% in vehicle VSMCs). Additionally, Flu significantly reversed the alterations of the VSMC cell cycle elicited by PDGF. The percentage of cells in G0/G1 increased to 53.8% ± 4.2%, while the cells in S phase decreased to 30.3% ± 2.8% when cotreated with 10^−5^ M Flu and 10 *µ*g/L PDGF (Figure 2). This data indicated that Flu inhibited cell proliferation by inducing cell cycle arrest at the G0/G1 phase.

### 3.3. PDGF Time-Dependently Inhibited LTCC*α*_1C_ Expression Which Could Be Prevented by Fluvastatin

**Figure 3:**
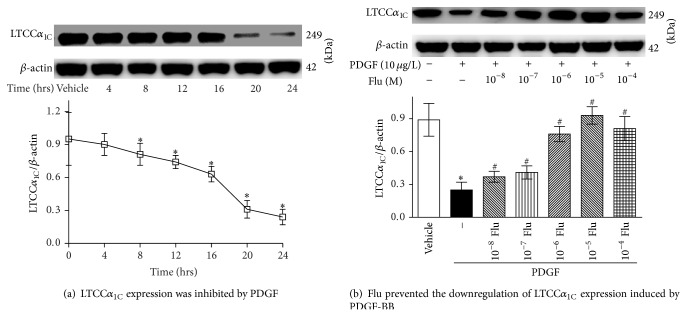
Effects of Flu on the levels of LTCC*α*_1C_ protein determined by western blot analysis. PDGF time-dependently inhibited LTCC*α*_1C_ expression, which could be prevented by fluvastatin. (a) Dose-effect response of PDGF stimulation on the LTCC*α*_1C_ expression in VSMCs. (b) Time-effect response of Flu on the LTCC*α*_1C_ expression incubated by PDGF. β-actin served as the loading control. Data was represented as means ± SE of 3 separate experiments conducted in triplicate. ^*^ *P* < 0.05 versus blank control and ^#^ *P* < 0.05 versus cells incubation with PDGF. LTCC*α*_1C_: L-type calcium channel *α*_1C_ subunit; Flu: fluvastatin; and PDGF: platelet derived growth factor.

To determine whether the VSMC phenotype switching was associated with the alterations in LTCC*α*_1C_ expression, the protein level of LTCC*α*_1C_ was determined by western blot analysis. PDGF suppressed the LTCC*α*_1C_ protein expression time-dependently and the peak effect occurred after PDGF stimulation for 24 h (both *P* < 0.01, Figure 3(a)). Exposure to PDGF for 24 h decreased LTCC*α*_1C_ protein expression by 74.7%, as compared with vehicle cells. In addition, fluvastatin dose-dependently reversed the downregulation of LTCC*α*_1C_ expression elicited by PDGF-BB. And the most efficient Flu concentration was at 10^−5^ M. LTCC*α*_1C_ protein expression was increased by 2.72-fold in VSMCs cotreated with PDGF and Flu, as compared to that in VSMCs incubated with PDGF (Figure 3(b)).

### 3.4. MAPK or ROCK Inhibition Mimicked the Statin Effects by Upregulating LTCC*α*_1C_ Expression

**Figure 4:**
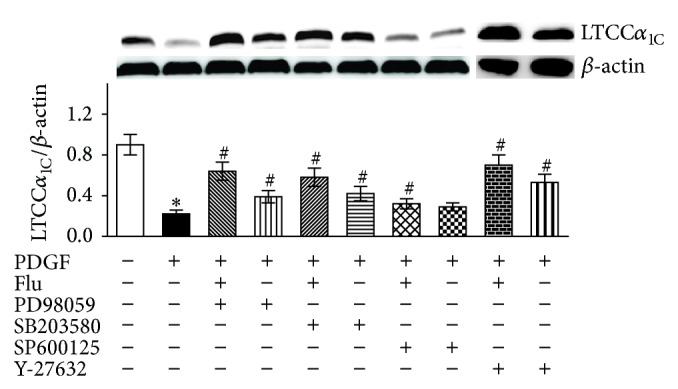
Effects of MAPK or ROCK inhibition on LTCC*α*_1C_ expression after PDGF stimulation in VSMCs. LTCC*α*_1C_ protein expression was evaluated by western blot analysis. Data was described as means ± SEM from three experiments performed in triplicate. ^*^ *P* < 0.05 versus blank control and ^#^ *P* < 0.05 versus PDGF stimulated cells. MAPK: mitogen activated protein kinase; ROCK: Rho associated protein kinase; LTCC*α*_1C_: L-type calcium channel *α*_1C_ subunit; Flu: fluvastatin; and PDGF: platelet derived growth factor.

To investigate a direct role of MAPK and RhoA pathway in the PDGF-mediated regulation of LTCC*α*_1C_ expression, the specific pharmacologic inhibitors, ERK1/2 inhibitor (PD98059, 20 *μ*mol/L), p38 MAPK inhibitor (SB203580, 10 *μ*mol/L), JNK inhibitor (SP600125, 50 *μ*mol/L), or Rho kinase inhibitor (Y27632, 10 *μ*mol/L), were pretreated for 1 h before PDGF administration. As shown in Figure 4, PD98059, SB203580, or Y27632 significantly attenuated but did not abolish the PDGF-induced downregulation of LTCC*α*_1C_ expression (both *P* < 0.01). Meanwhile, inhibiting ERK, p38 MAPK, or ROCK activation largely augmented the upregulation effect of fluvastatin (*P* < 0.01). However, blockade of JNK pathway had no effect on LTCC*α*_1C_ expression. Together, these results indicated that the RhoA, ERK1/2, and p38 MAPK pathway was involved in the regulation of LTCC*α*_1C_ by fluvastatin in VSMCs stimulated by PDGF.

### 3.5. Effect of Fluvastatin on PDGF-Induced Activation of MAPK

**Figure 5:**
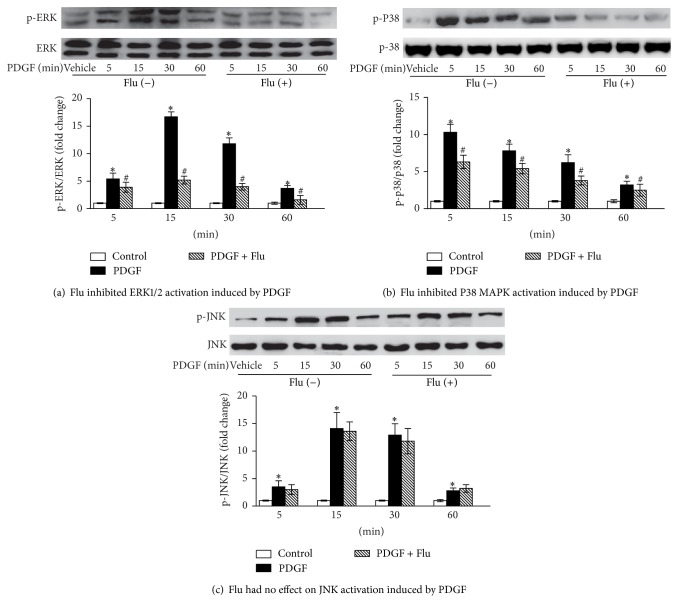
Effect of Flu on PDGF-induced phosphorylation of ERK1/2, p38 MAPK, and JNK. Phosphorylation of ERK1/2, p38 MAPK, and JNK was detected by western blotting using specific antibodies. Total ERK1/2, p38 MAPK, and JNK proteins were used as internal controls. Quantification of band intensities from three independent experiments was determined by densitometry. Data was described as means ± SEM from three experiments performed in triplicate. ^*^ *P* < 0.05 versus blank control and ^#^ *P* < 0.05 versus cells incubation with PDGF. Flu: fluvastatin; PDGF: platelet derived growth factor. ERK1/2: extracellular signal regulated kinase; MAPK: mitogen activated protein kinase; and JNK: c-Jun N-terminal kinase.

VSMCs are known to acquire proliferative characteristics through the MAPK pathway, of which extracellular signal regulated kinase (ERK) 1/2, p38 MAP kinase, and c-Jun N-terminal kinase (JNK) are key components that play critical roles in the VSMCs proliferation and cell cycle progression. For this purpose, we tested whether Flu affected the activity of ERK1/2, p38 MAPK, and JNK in response to PDGF. VSMCs were cultured with or without fluvastatin (10^−5^ M) for 24 h and then treated with PDGF (10 *μ*g/L) for 5, 15, 30, and 60 min. Upon PDGF-BB stimulation for 5–60 min, ERK1/2, p38 MAPK, and JNK activation was dramatically increased in VSMCs, and cotreatment with PDGF and 10^−5^ M Flu significantly inhibited ERK1/2 and p38 MAPK activation in a time-dependent manner (Figures 5(a)-5(b)). However, activation of JNK was not affected by Flu in PDGF-stimulated VSMCs (Figure 5(c)). Additionally, the total ERK1/2, p38 MAPK, and JNK levels were not altered by Flu. Collectively, these data indicated that Flu could suppress the phosphorylation of ERK1/2 and p38 MAPK elicited by PDGF.

### 3.6. Regulation of Membrane Localization and Activation of RhoA by Fluvastatin

**Figure 6:**
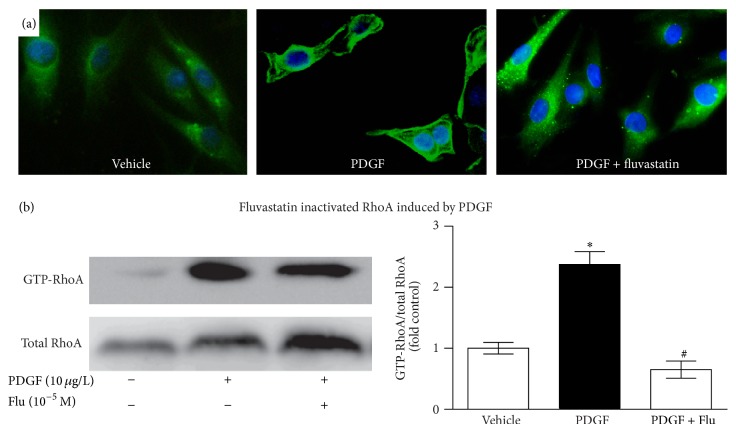
Regulation of membrane-associated RhoA by PDGF in the absence and presence of fluvastatin. (a) Localization of RhoA was determined by immunofluorescent staining. Distribution of RhoA (green) in VSMCs. Nuclei were stained with DAPI (blue). (b) RhoA activation by PDGF in the absence and presence of fluvastatin. RhoA activation was evaluated by rate of GTP-Rho (active form) and total RhoA. Quantification of band intensities from three independent experiments was determined by densitometry. Data was described as means ± SEM from three experiments performed in triplicate. ^*^ *P* < 0.05 versus blank control and ^#^ *P* < 0.05 versus cells incubation with PDGF. RhoA: ras homolog family member A; Flu: fluvastatin; and PDGF: platelet derived growth factor.

We next evaluated whether Flu could inhibit the activation of the small G protein Rho caused by PDGF. In the quiescent state, Rho binds to GDP and resides in the cytosol. On activation, GDP-Rho is converted to GTP-Rho and translocated to the membrane. In VSMCs by indirect immunofluorescent staining, we observed a weak diffuse cytoplasmic RhoA staining in unstimulated cells (Figure 6(a)). Treatment with PDGF changed staining pattern from diffuse cytosolic to membrane localized, indicating activation of RhoA. This change in RhoA distribution was blocked by fluvastatin. Additionally, as indicated by pull-down assays, GTP-bound Rho levels increased 2.37-fold after incubation with PDGF for 24 hours, which were attenuated by pretreatment with fluvastatin (Figure 6(b)).

## 4. Discussion

In the present study, we found that (1) PDGF stimulation inhibited LTCC*α*_1C_ expression in VSMCs by activating RhoA, ERK1/2, and p38 MAPK pathways; (2) fluvastatin promoted a more differentiated VSMC phenotype concurrent with the upregulation of LTCC*α*_1C_ expression via inactivating RhoA, ERK1/2, and p38 MAPK pathways; and (3) LTCC*α*_1C_ was downregulated in the proliferating smooth muscle cells. It was a contractile phenotype marker. To the best of our knowledge, our investigation is the first to report the direct effect and the underlying mechanism of fluvastatin treatment on LTCC*α*_1C_ expression in vitro. Our results indicated that PDGF suppressed LTCC*α*_1C_ expression, which was similar to the reports that L-type Ca^2+^ channel was lost when quiescent VSMCs underwent a phenotypic switch to the proliferating/synthetic state [16, 17]. Alternatively, our results were partly substantiated by the investigations that fluvastatin treatment prevented left atrium LTCC*α*_1C_ subunit downregulation in atrial tachycardia dogs [18]. Our present findings seemingly contradicted our previous investigations that, in hypertrophic pulmonary arteries induced by monocrotaline, atorvastatin downregulated LTCC*α*_1C_ expression [19]. The possible explanation for this discrepancy might be the lacking of self-homeostatic regulation, neurohumoral regulation, and vascular tone in vitro. This argument was supported by those reports that the expression of the pore-forming *α*_1C_ subunit of the CaV1.2 channel was elevated in arteries of hypertensive animals compared to age-matched normotensive animals [20, 21] while, in proliferating VSMCs in vitro, the LTCC*α*_1C_ expression was downregulated [22].

The mechanistic insights into the regulating the phenotypic switch of VSMC have been intensely studied. Our data reveals that fluvastatin attenuates PDGF-induced LTCC*α*_1C_ expression, at least in part, by inhibiting ERK1/2 and p38 MAPK activation. PDGF is known to induce cell proliferation via a pathway involving PKC and small GTPases (Rho, Rac) [23]; ROCK acts as an upstream regulator leading to the activation of the MAPKs family, including p38 MAPK and ERK [24]. PDGF represses the characteristic VSMC gene expression by activating ERK1/2 and p38 MAPK pathways in cultured VSMCs [5, 25]. HMG CoA catalyses the conversion of HMG CoA to mevalonate which can then be further metabolized to cholesterol. However, mevalonate is also the precursor of the isoprenoids, farnesyl pyrophosphate (FPP), and geranylgeranyl pyrophosphate (ggPP) which plays a key role in the lipid modification of small G proteins, such as Ras and Rho. Depletion of isoprenoids by statins results in accumulation of nonfunctional Rho GTPases in the cytoplasm [26]. Statins therefore not only block cholesterol synthesis but also may inhibit the Ras and Rho signaling pathways. Considering that MAPK was the downstream molecules of isoprenoid pathway, it is not surprising that fluvastatin can upregulate LTCC*α*_1C_ expression via inhibiting MAPK signal pathway. However, it should be noted that the inactivation of Rho or MAPK could not fully abolish the PDGF-induced downregulation of LTCC*α*_1C_ expression, suggesting that other signaling pathway(s) might be involved in the regulation of LTCC*α*_1C_ by PDGF.

In conclusion, our novel findings indicated that fluvastatin prevented the PDGF-induced downregulation of LTCC*α*_1C_ expression through the suppression of RhoA, ERK1/2, or p38 MAPK signaling. Our data is encouraged to unravel why calcium channel blockers are effective in uninjured arteries and at the early stages of disease but subsequently lose efficacy as the disease progresses and VSMCs become progressively more dedifferentiated. Furthermore, our findings indicate that statins induce a more differentiated VSMC phenotype paralleling the increased L-type calcium channels expression, which provide a rationale for the synergistic effects of statins and calcium channel blockers to lower blood pressure in hypertensive patients [27, 28]. Considering that posttranslation modifications can alter the function of ion channel without requiring the changes in protein expression, patch-clamp experiments will be necessary to further verify the role of fluvastatin in calcium channel properties. Additionally, further studies are needed to clarify the involvement of Rac1 and Cdc42 in the upregulation effects of fluvastatin on LTCC*α*_1C_ expression by PDGF.
