# Corrigendum to “Fluvastatin Upregulates the *α*_1C_ Subunit of CaV1.2 Channel Expression in Vascular Smooth Muscle Cells via RhoA and ERK/p38 MAPK Pathways”

**DOI:** 10.1155/2024/9875935

**Published:** 2024-06-22

**Authors:** Qiu-Fang Ouyang, Ying Han, Zhi-Hong Lin, Hong Xie, Chang-Sheng Xu, Liang-Di Xie

**Affiliations:** ^1^ Fujian Hypertension Research Institute The First Affiliated Hospital of Fujian Medical University, Fujian 350005, China; ^2^ Ultrasound Department The Second Affiliated People's Hospital Fujian University of Traditional Chinese Medicine, Fuzhou, Fujian 350003, China

In the article titled “Fluvastatin Upregulates the *α*_1C_ Subunit of CaV1.2 Channel Expression in Vascular Smooth Muscle Cells via RhoA and ERK/p38 MAPK Pathways” [1], the corresponding author's email address is no longer in use. The correct email address is shown above. In addition to this, the western blots for Figure 1 in the *β*-actin lane were reported to be a rotation of the lanes presented in Figure 3(b) for *β*-actin. The authors have explained that the error occurred during manuscript revisions. The corrected Figure 1 is shown below.

## Figures and Tables

**Figure 1:**
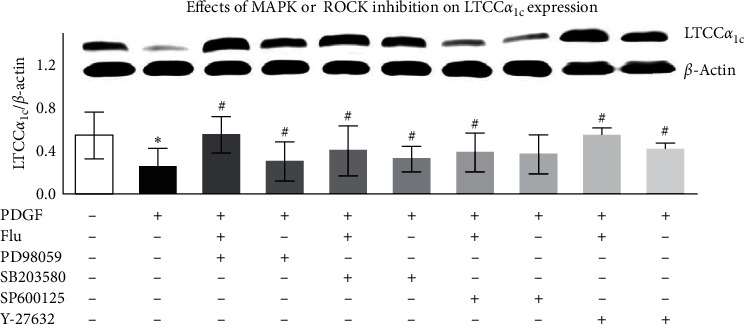
Effects of MAPK or ROCK inhibition on LTCC*α*_1C_ expression after PDGF stimulation in VSMCs. LTCC*α*_1C_ protein expression was evaluated by western blot analysis. Data was described as means ± SEM from three experiments performed in triplicate.  ^*∗*^*P*  < 0.05 versus blank control and  ^#^*P* < 0.05 versus PDGF stimulated cells. MAPK: mitogen-activated protein kinase; ROCK: Rho-associated protein kinase; LTCC*α*_1C_: L-type calcium channel *α*_1C_ subunit; Flu: fluvastatin; and PDGF: platelet-derived growth factor.

## References

[1] Ouyang Q.-F., Han Y., Lin Z.-H., Xie H., Xu C.-S., Xie L.-D. (2014). Fluvastatin Upregulates the *α*_1C_ Subunit of CaV1.2 Channel Expression in Vascular Smooth Muscle Cells via RhoA and ERK/p38 MAPK Pathways. *Disease Markers*.

